# Comprehensive Quantification of Oligoasthenozoospermia Induced by Obesity, Reproductive Toxicants, and Their Combination in Rat Models

**DOI:** 10.1111/andr.70171

**Published:** 2026-01-23

**Authors:** Yunlong Yao, Xi Zhang, Lingru Li, Yunzhi Zhang, Shuo Huang, Yulin Zong, Mingrui Li, Fufangyu Zhao, Linghui Zhu, Tianxing Li, Wenlong Sun, Mingyan Shao, Yanfei Zheng

**Affiliations:** ^1^ School of Traditional Chinese Medicine Beijing University of Chinese Medicine Beijing China; ^2^ National Institute of Traditional Chinese Medicine Constitution and Preventive Medicine Beijing University of Chinese Medicine Beijing China; ^3^ School of Life Sciences and Medicine Shandong University of Technology Shandong China

**Keywords:** gut–testis axis, obesity, oligoasthenozoospermia, reproductive toxicants, transcriptomics

## Abstract

**Background:**

Oligoasthenozoospermia is a leading cause of male infertility and has been increasingly associated with the global surge in obesity and exposure to reproductive toxicants. Despite extensive research on each factor individually, their combined pathological effects remain poorly understood.

**Objectives:**

This study aimed to investigate how obesity and reproductive toxicants independently and synergistically impair male reproductive function by establishing and comparing rat models of oligoasthenozoospermia induced by each factor and their combination.

**Materials and Methods:**

We constructed three etiologically distinct oligoasthenozoospermia male Sprague–Dawley rat models: a reproductive toxicity model induced by glycosides of *Tripterygium wilfordii* Hook. f. (GTW), a metabolic dysfunction model induced by a high‐fat diet (HFD), and a combined model (HFD + GTW). Animals were randomly assigned and subjected to 12 weeks of treatment. Body weight, metabolic indices, serum sex hormone levels, sperm quality parameters, and histopathological analysis of the testes and epididymis, as well as gut microbiota composition and testicular transcriptome profiles, were systematically evaluated. We also performed quantitative real‐time polymerase chain reaction.

**Results:**

Both GTW and HFD independently impaired reproductive function, leading to decreased sperm count and motility, hormonal disturbances, and moderate testicular damage. The combined model exhibited significantly exacerbated reproductive impairment, including extensive spermatogenic cell loss, disrupted testicular architecture, and the lowest sperm quality indices. Multiomics analysis revealed coordinated alterations in gut microbiota composition and testicular transcriptomes, suggesting crosstalk between metabolic and inflammatory signaling pathways. Quantitative real‐time polymerase chain reaction confirmed these transcriptomic patterns, showing upregulation of *Ahnak*, *C1r*, *S1pr1*, and *Steap4*, alongside downregulation of *Alkbh7*, *Tbpl1*, *Tent5b*, and *Ldhal6b* in the models.

**Conclusion:**

This study successfully establishes reliable rat models that mimic both individual and combined etiologies of oligoasthenozoospermia. The interaction between obesity and GTW‐induced reproductive toxicity aggravates testicular injury through metabolic disruption and inflammatory pathways, offering an integrative platform for mechanistic and therapeutic research.

## 1 Introduction

Infertility is a major global reproductive health issue, affecting approximately 8%–12% of couples of reproductive age, with male factors directly or indirectly contributing to nearly 60% of cases [1]. Among male infertility cases, more than 40% are attributed to seminal abnormalities [2]. Clinically, oligoasthenozoospermia, characterized by reduced sperm concentration and impaired motility [2], is one of the most common seminal abnormalities associated with male infertility. Given its prevalence and contribution to the global health burden, understanding the underlying mechanisms of oligoasthenozoospermia is essential for developing effective therapeutic interventions.

Oligoasthenozoospermia arises from multiple contributing factors and has a complicated pathogenic basis. In recent years, the growing prevalence of obesity has made it an increasingly important factor affecting oligoasthenozoospermia [3]. Men with obesity have a significantly higher risk of developing oligoasthenozoospermia than those with normal body weight [4, 5, 6]. The underlying mechanisms include endocrine dysregulation—characterized by reduced testosterone (T) and elevated estrogen levels that inhibit spermatogenesis [7, 8], as well as scrotal fat accumulation, increased testicular temperature, chronic inflammation, and oxidative stress, all of which impair spermatogenic function [9, 10]. Exposure to reproductive toxicants represents another major cause of oligoasthenozoospermia. Environmental pollutants (e.g., heavy metals, pesticides, and tobacco smoke) and certain drugs or natural compounds (such as glycosides of *Tripterygium wilfordii* Hook. f., GTW) can impair male reproductive function through multiple pathways; they can directly damage the testicular microenvironment, disrupt spermatogenesis, or induce oxidative stress and hormonal imbalance. These mechanisms collectively result in decreased sperm count (oligozoospermia), impaired motility (asthenozoospermia), and morphological abnormalities, ultimately diminishing male fertility [11, 12, 13]. Clinically, obesity‐ or reproductive toxicity‐induced oligoasthenospermia are common subtypes. GTW, bioactive components extracted from *T. wilfordii*, are widely used in the clinical management of autoimmune and inflammatory diseases such as rheumatoid arthritis, systemic lupus erythematosus, and nephrotic syndrome due to their strong anti‐inflammatory and immunosuppressive properties [14, 15]. However, extensive experimental and clinical evidence has confirmed that GTW induces reproductive toxicity in males. GTW exposure causes testicular histopathological abnormalities, decreases testis weight, impairs spermatogenesis, and reduces sperm motility and viability through mechanisms involving oxidative stress, apoptosis, and testicular microenvironment disruption. Accordingly, GTW is frequently used as a classical agent to establish reproductive toxicity models in preclinical research [16].

The concurrent rise in obesity and widespread consumption of high‐fat diet (HFD) has created a setting wherein metabolic dysfunction and exposure to reproductive toxicants may coexist. Clinically, many patients receiving GTW therapy also present with metabolic syndrome or are overweight, suggesting potential interactions between these risk factors [17, 18]. Metabolic stress may exacerbate chemical toxicity by amplifying oxidative and inflammatory responses, further impairing spermatogenic capacity [19]. Therefore, investigating the combined effects of HFD and GTW exposure is both scientifically and clinically significant, as it mirrors real‐world conditions of rising obesity prevalence alongside continued GTW use.

Thus, establishing reliable animal models is crucial for elucidating the mechanisms of oligoasthenozoospermia and identifying potential therapeutic approaches. In this study, we developed three rat models of oligoasthenozoospermia: a GTW‐induced model to represent reproductive toxicity (GTW model), an HFD‐induced model to mimic obesity (HFD model), and a combined HFD + GTW model to simulate the coexistence of metabolic dysfunction and reproductive toxicity (HFD + GTW model). We further integrated 16S ribosomal RNA (rRNA) gene sequencing with transcriptomic profiling to explore disease mechanisms driven by different etiological factors. This study aims to elucidate the pathophysiological processes underlying various forms of oligoasthenozoospermia and to identify potential molecular targets for clinical management of male infertility.

## 2 Materials and Methods

### 2.1 Animals and Experimental Design

A total of 48 specific pathogen‐free (SPF) male Sprague–Dawley (SD) rats (weight: 200 ± 20 g; Beijing Vital River Laboratory Animal Technology Co., Ltd., SCXK2021‐0006; Beijing, China) were housed under SPF conditions with a 12‐h light/dark cycle, controlled temperature (21–25°C), and humidity (40%–70%). After a 1‐week acclimatization period, the rats were randomly assigned to the following four groups: control, GTW, HFD, and HFD + GTW. The control rats were fed a standard diet. The GTW model rats were fed a standard diet and administered 60 mg/kg GTW; this dose was selected based on previous reports demonstrating the reliability of this regimen in inducing testicular injury and oligoasthenozoospermia in rats without causing overt systemic toxicity [16]. The HFD model rats were fed HFD. The HFD + GTW model rats were fed HFD and administered 60 mg/kg GTW. The rats were fed HFD or a standard diet for 12 weeks, during which GTW administration was initiated at Week 9 via daily gavage for 4 consecutive weeks. The HFD consisted of 45% kcal from fat, 35% from carbohydrate, and 20% from protein, totaling 4.73 kcal/g (Changzhou SYSE. Bio‐tec Co., Ltd., Changzhou, China). All feeds were purchased from Jiangsu Synergetic Biological Co., Ltd. and stored at 4°C. GTW (product code: 100007987379) was purchased from Fudan‐Fukang Pharmaceutical Co., Ltd. All experimental procedures were approved by the Ethics Committee for Laboratory Animals at Beijing University of Chinese Medicine (approval number: BUCM‐2024080504‐3077).

### 2.2 Body Weight and Phenotypic Assessments

All rats were weighed at 7‐day intervals on a calibrated electronic balance (accuracy ± 0.1 g). At the end of the study, the rats were anesthetized via intraperitoneal injection of 1% pentobarbital, and their body length (from the nose tip to the anus) was measured. Their body mass index (BMI) and Lee's index were calculated using the following equation:

$$\mathrm{BMI}=\mathrm{body}\mathrm{weight}/{\left(\mathrm{body}\mathrm{length}\right)}^{2}$$

$${\mathrm{Lee}}^{\prime }s\mathrm{index}=\left(\mathrm{body}\mathrm{weigh}t^{1/3}/\mathrm{body}\mathrm{length}\right).$$

The testes and epididymides were collected after removing the surrounding adipose tissues, weighed, and then photographed for documentation. The testis index (testis weight/body weight) and epididymal fat index (epididymal fat weight/body weight) were subsequently calculated.

### 2.3 Biochemical Assessments

The serum samples were collected via the abdominal aorta and placed in plain tubes. The levels of serum high‐density lipoprotein cholesterol (HDL‐C), low‐density lipoprotein cholesterol (LDL‐C), triglycerides (TG), and total cholesterol (TC) were detected by using the assay kit (Nanjing Jiancheng Bioengineering Institute, China) according to the protocol provided by the manufacturer. The liver function parameters, including alanine aminotransferase (ALT) and aspartate aminotransferase (AST), and kidney function parameters, including blood urea nitrogen (BUN) and creatinine (Cr), were analyzed by using commercially available assay kits manufactured by Nanjing Jiancheng Bioengineering Institute as per the manufacturer's protocols.

### 2.4 Measurement of the Serum Sex Hormone Levels

The serum testosterone (T), estradiol, luteinizing hormone (LH), follicle‐stimulating hormone (FSH), and prolactin levels were determined by using an enzyme‐linked immunosorbent assay (ELISA) kit (Wuhan Youke Protein Biotechnology) in accordance with the manufacturer's instructions.

### 2.5 Sperm Quality Evaluation

The head of the epididymis was dissected and incubated in 1 mL of M199 medium at 37°C to allow sperm dispersion. A 10‐µL aliquot was then placed on a counting chamber. Sperm concentration and motility were assessed by using a sperm analysis system (WL‐9000; Weili Technology Co., Ltd., Beijing) at 37°C. Four random fields were analyzed within 2 min. The parameters recorded included the sperm concentration (10⁶/mL), progressive motility (%), sperm count (10⁶), and total motility (%).

### 2.6 Hematoxylin and Eosin Staining

The testis and epididymis from the rats were fixed in Bouin's solution for 24 h, dehydrated in a graded series of ethanol, embedded in paraffin, and sectioned at 3‐µm thickness. After dewaxing and hydration, the sections were washed under running water for 5 min and immersed in deionized water for 2 min. Hematoxylin and Eosin (H&E) staining was then performed, followed by dehydration in 95% and absolute ethanol, clearing with xylene, and mounting with resin. Histological observations were performed by using a light microscope (Primo Star, Carl Zeiss). Johnsen's score examination was performed in 100 seminiferous tubules per animal (a total of 700 seminiferous tubules in each group) as described previously (Table S1).

### 2.7 16S rRNA Gene Sequencing

Total microbial DNA was extracted from fecal samples by using the FastPure Stool DNA Isolation Kit. Integrity and concentration were confirmed by 1% agarose gel electrophoresis and NanoDrop 2000. The V3–V4 regions of the 16S rRNA gene were amplified with barcoded primers 338F and 806R. The PCR reactions were run with 27 cycles, and each sample was amplified in triplicate for reproducibility. The products were purified and quantified with Qubit 4.0. Library construction was performed using the NEXTFLEX Rapid DNA‐Seq Kit, and sequencing was performed on the Illumina NextSeq 2000 platform (Shanghai Meiji Biomedical Technology Co., Ltd.).

Raw reads were quality‐filtered with fastp and merged using FLASH. High‐quality sequences were clustered into OTUs using UPARSE at 97% similarity, and the chimeras and non‐bacterial sequences were removed. The OTU tables were rarefied to 20,000 reads per sample with > 99% Good's coverage. Taxonomic annotation was performed using RDP Classifier against the SILVA database (v138) at 70% confidence. PICRUSt2 was used for functional prediction. Alpha diversity indices (sobs, ace, and chao) were calculated using Mothur, and Wilcoxon tests were performed. Beta diversity was assessed via principal component analysis (PCA) and ANOSIM. Linear discriminant analysis Effect Size (LEfSe; LDA > 2, *p* < 0.05) identified differentially abundant taxa. Distance‐based redundancy analysis (db‐RDA) examined the influence of clinical parameters on the gut microbiota.

### 2.8 RNA Sequencing Analysis of Testicular Tissue

Total RNA was extracted from liquid nitrogen‐ground testis tissues using TRIzol reagent as per the manufacturer's protocol. RNA concentration and purity were assessed by NanoDrop; integrity was confirmed by agarose gel electrophoresis and Agilent 5300 Bioanalyzer. Only high‐quality RNA (RIN ≥ 6.5, OD260/280 at 1.8–2.2) was used for library construction. mRNA was enriched with Oligo (dT) beads, fragmented to approximately 300 bp, and reverse‐transcribed into cDNA. The libraries were prepared using the Illumina NovaSeq Reagent Kit, including end repair, A‐tailing, adapter ligation, and PCR amplification, quantified with Qubit 4.0, and sequenced on the NovaSeq Xplus platform.

Raw data were quality‐controlled using fastp to remove low‐quality reads and adapters. The clean reads were then aligned to the reference genome using HISAT2 and assembled using StringTie. The gene expression was normalized with RSEM, and differential expression analysis was conducted using DESeq2, with the thresholds of FDR < 0.05 and |log2FC| ≥ 1.5. To identify the shared and unique transcriptomic alterations induced by different pathogenic factors, a Venn diagram was prepared across the three models. For visualization, the heatmap was constructed based on the top 20 representative DEGs commonly identified across all three model groups. These genes were selected in accordance with their ranking by *p* value (*p* < 0.05), representing the most significantly altered and biologically relevant transcripts among the shared DEGs. To further explore the potential interaction between gene expression and microbial dysbiosis, Spearman correlation analysis was conducted among these 20 key DEGs and the representative bacterial genera. Functional enrichment analysis of differentially expressed genes (DEGs) was conducted using Gene Ontology (GO) and Kyoto Encyclopedia of Genes and Genomes (KEGG) databases. GO annotations were performed with Diamond, and KEGG enrichment with KOBAS. Significance was assessed by Fisher's exact test with FDR correction (adjusted *p* < 0.05).

### 2.9 Quantitative Real‐Time Polymerase Chain Reaction

The selection of genes for quantitative real‐time polymerase chain reaction (qRT‐PCR) validation was based on the clustering results from the transcriptomic heatmap and categorized into four functional groups: obesity‐related, spermatogenesis‐related, oxidative stress‐related, and genes with no prior research in the related fields. Two representative genes were selected from each functional group for validation. Total RNA was extracted from testicular tissues using TRIzol reagent (Servicebio, China) in accordance with the manufacturer's instructions. RNA concentration and purity were assessed by using the NanoDrop 2000 spectrophotometer (Servicebio). Reverse transcription was performed using the PrimeScript RT Reagent Kit (Servicebio) to synthesize complementary DNA (cDNA) from 1 µg of total RNA. Quantitative real‐time PCR was conducted on the CFX96 Real‐Time PCR System (Servicebio) using SYBR Premix Ex Taq II (Vazyme, China). β‐Actin served as an internal reference gene. The thermal cycling conditions were as follows: 95°C for 30 s, followed by 40 cycles of 95°C for 15 s and 60°C for 30 s. Each reaction was performed in triplicate. The relative mRNA expressions of target genes were calculated by using the 2^^−ΔΔCt^ method. The primer sequences were synthesized by Sangon Biotech (Servicebio), and are listed in Table S2.

### 2.10 Data Preprocessing and Statistical Analysis

Quantitative data were presented as the mean ± SD. One‐way analysis of variance (anova) was applied for comparisons among multiple groups. *p* < 0.05 was considered to indicate statistical significance. Analyses were performed using GraphPad Prism 10.4.0 (GraphPad Software).

## 3 Results

### 3.1 GTW, HFD, and HFD + GTW Exposures Differentially Affected Body Weight, Lipid Profiles, and Systemic Organ Function in Rats

**FIGURE 1:**
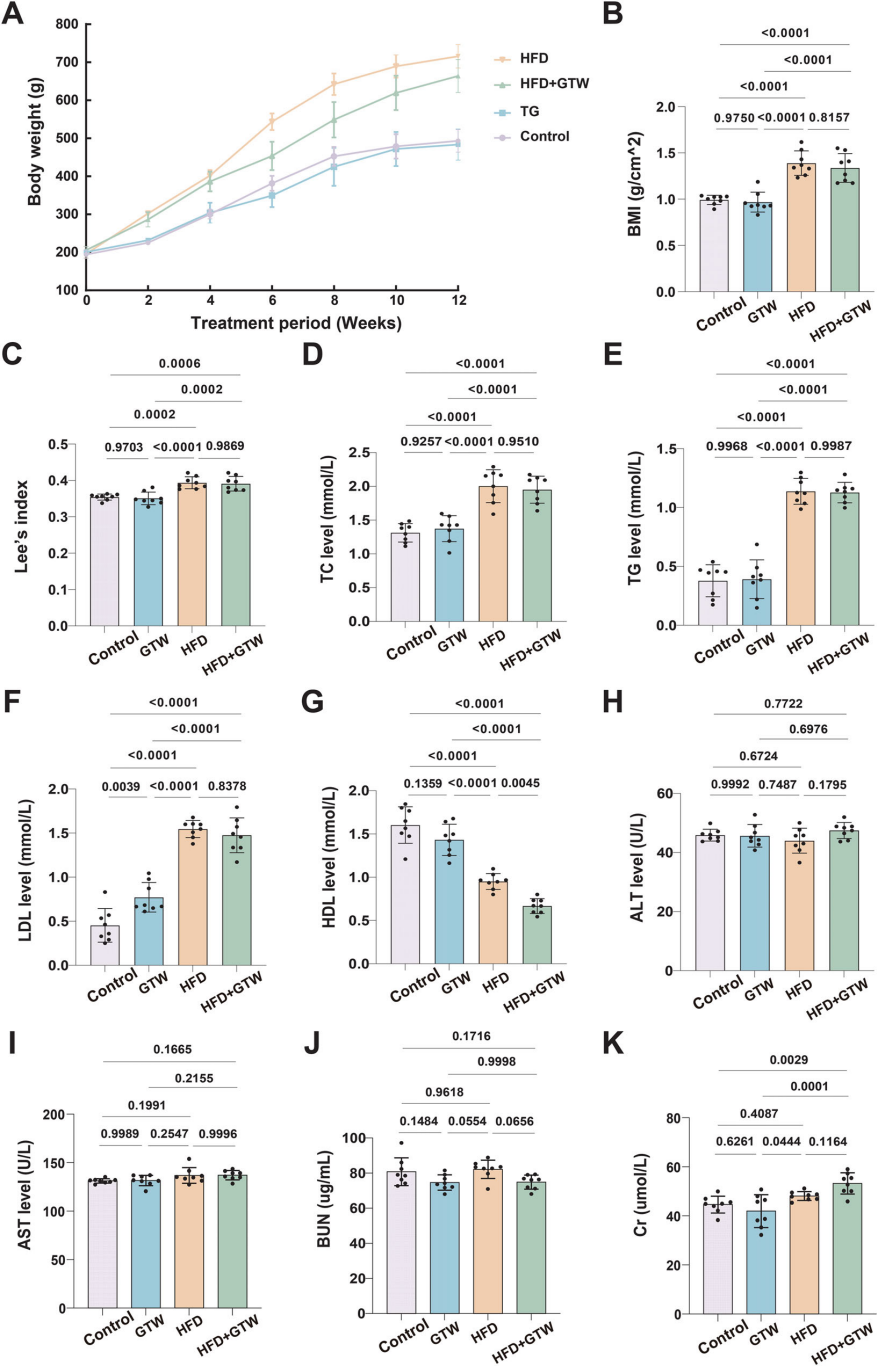
Effects of HFD and GTW exposure on body composition, lipid profile, and organ function. (A) Weekly body weight progression over 12 weeks. (B) Body mass index (BMI) and (C) Lee's index were significantly increased in HFD‐fed rats (HFD and HFD + GTW model) when compared with the control and GTW models. (D–G) Serum lipid parameters (i.e., TC, TG, LDL‐C, and HDL‐C) revealed dyslipidemia in the HFD and HFD + GTW models. (H, I) Liver function markers (ALT and AST) exhibited no significant differences across the groups. (J, K) Kidney function analysis revealed elevated serum Cr in the HFD + GTW model, whereas BUN level remained unchanged. Data are presented as the mean ± SEM. One‐way anova with post hoc test (*n* = 8/group).

Compared with rats in the control group, those in the HFD and HFD + GTW models showed significant increases in body weight, BMI, Lee's index, and epididymal fat index (Figures 1A–C and S1C). In contrast, the testis index was significantly decreased in both HFD and HFD + GTW models compared with the control group (Figure S1B). Moreover, the HFD and HFD + GTW models exhibited significantly higher body weight, BMI, and Lee's index than the GTW model (Figure 1A–C). Obesity‐related measures were similar between the HFD and HFD + GTW models, reflecting comparable degrees of diet‐induced obesity. Consistent with these trends, the epididymal fat index was substantially increased in the HFD models. The HFD + GTW model exhibited a significantly higher fat index than the GTW model, whereas the increase in the HFD model compared with the GTW model was not statistically significant. No significant difference in fat index was noted between the HFD and HFD + GTW models (Figure S1C). Relative testis weights were significantly lower in both obese models, with the HFD and HFD + GTW models displaying reduced testis index values compared with the GTW model. No significant testis index difference was noted between the HFD and HFD + GTW models, indicating that obesity (regardless of reproductive toxicity) led to a comparable reduction in testicular mass (Figure S1B).

A hallmark of obesity‐related oligoasthenozoospermia is dyslipidemia, characterized by elevated blood lipids and altered hormone levels. Compared with the control group, the levels of TC, TG, and LDL‐C were significantly increased in the HFD and HFD + GTW models (Figure 1D–F), whereas HDL‐C was significantly decreased (Figure 1G). Interestingly, although GTW model rats were also fed a standard diet, their LDL‐C levels were significantly elevated compared with the control group (Figure 1F), while no significant differences were observed in TC, TG, and HDL‐C levels (Figure 1E,G,H). The HFD and HFD + GTW models showed significantly higher TC, TG, and LDL‐C levels and lower HDL‐C levels compared with the GTW model, but no significant differences were observed in TC, TG, and LDL‐C levels between the HFD and HFD + GTW models except for HDL‐C, suggesting that GTW co‐exposure did not further worsen HFD‐induced lipid abnormalities.

No significant differences were observed in AST or ALT levels across the groups, suggesting preserved hepatic function (Figure 1H,I). However, serum Cr levels were significantly elevated in the HFD + GTW model compared with both the control group and GTW model, while BUN and Cr levels remained comparable among the other models, suggesting preserved renal function (Figure 1J,K).

### 3.2 GTW, HFD, and HFD + GTW Exposures Caused Varying Degrees of Reproductive Hormone Disruption, Sperm Impairment, and Testicular Histopathology

**FIGURE 2:**
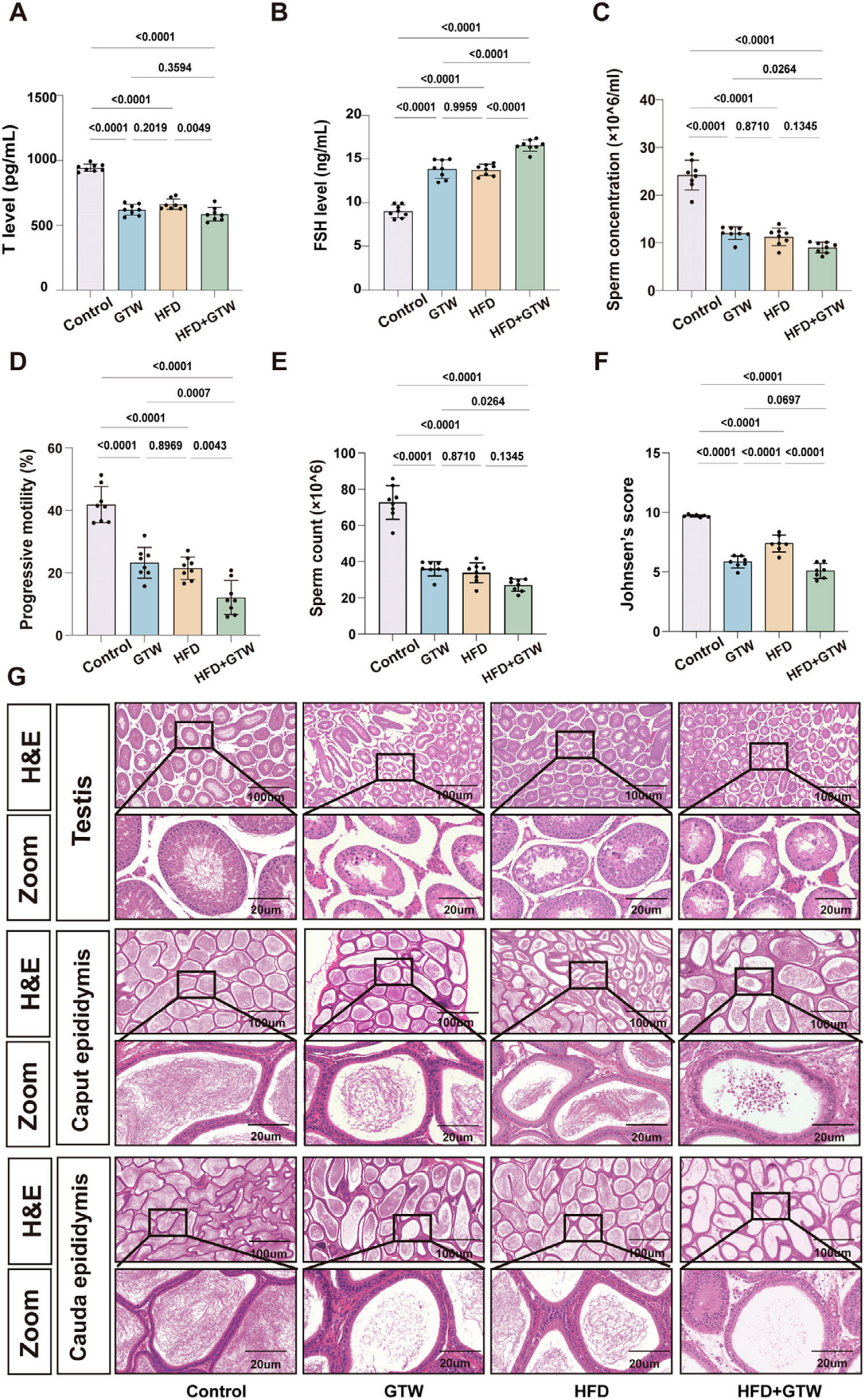
Reproductive hormone disruption, impaired sperm quality, and testicular histopathology in rat models. (A, B) The serum levels of testosterone (T) were significantly reduced in all model groups, whereas the FSH levels were elevated, with the HFD + GTW model displaying the most pronounced changes. (C–E) Sperm concentration, progressive motility, and sperm count were markedly reduced in all models. (F) Johnsen's scores, reflecting the degree of spermatogenic integrity, were significantly reduced in the GTW and HFD + GTW models, indicating substantial testicular damage (*n* = 7/group). (G) Representative H&E‐stained sections of testis, caput epididymis, and cauda epididymis (upper row: scale bar = 100 µm; lower row: scale bar = 20 µm; *n* = 7/group). The control group rats exhibited intact seminiferous tubules and sperm‐rich epididymal lumens. The HFD + GTW model showed severe testicular architectural disruption, loss of spermatogenic layers, and reduced epididymal sperm density. Data are presented as the mean ± SEM. One‐way anova with post hoc test (*n* = 8/group).

Abnormalities in the sex hormone levels directly affect sperm production, development, and function. To assess endocrine changes, serum sex hormones were quantified. The T and LH levels were significantly decreased in all three models compared with the control group, indicating that both obesity and reproductive toxicity suppress T and LH levels (Figures 2A and S1D). In contrast, FSH levels were significantly elevated in all models (Figure 2B). No significant changes were observed in estradiol or prolactin among the models (Figure S1E,F). Further comparison revealed that the T and LH levels were significantly lower in the HFD + GTW model than in the HFD model, while no notable differences were found between the GTW and HFD models or between the GTW and HFD + GTW models (Figures 2A and S1D), suggesting that combined exposure may exert additive or synergistic effects on hormonal regulation. The FSH levels were significantly higher in the HFD + GTW model than in the GTW and HFD models, with no significant differences observed between the GTW and HFD models (Figure 2B). These findings suggest that the co‐exposure to HFD and GTW synergistically exacerbated HPG axis disruption in the HFD + GTW model, indicating significantly greater effects than either single intervention.

Sperm concentration and motility were significantly lower in all models than in the control group, with values falling below the World Health Organization reference thresholds for oligoasthenozoospermia (< 16 × 10⁶/mL for concentration, < 30% for progressive motility). The HFD + GTW model exhibited the lowest sperm concentration and motility across all experimental models (Figure 2C,D), indicating that co‐exposure to obesity and reproductive toxicity synergistically exacerbated testicular injury. Total sperm count and motility were significantly reduced in all models compared with the control group, further confirming the occurrence of oligoasthenozoospermia. The HFD + GTW model displayed the most severe impairments, with total sperm count and motility declining to the lowest levels among all experimental conditions (Figures  2E and S1G).

H&E staining of testicular and epididymal tissues revealed that the seminiferous tubules in the control group displayed normal morphology, with well‐organized spermatogenic cells, clearly defined spermatogenic layers, and abundant mature spermatozoa. The epididymal tubules in all segments were densely arranged and heavily filled with spermatozoa. In the HFD model, mild pathological changes were observed, including partial dilation of the seminiferous tubule lumens, slight exfoliation and disorganization of germ cells, and decreased sperm content in the epididymal lumen. The overall architecture remained comparatively intact, although the spermatogenic layers appeared slightly disrupted. In contrast, both GTW and HFD + GTW models exhibited severe testicular and epididymal lesions. The seminiferous tubules were markedly dilated and irregular, with extensive germ cell exfoliation, disrupted or absent spermatogenic layers, and only a few residual cells along the basement membrane. Epididymal tubules showed drastically reduced sperm content, the presence of exfoliated and degenerative cells, and disarrayed cilia. Among all models, the HFD + GTW model exhibited the most extensive tissue damage (Figure 2G). Johnsen's scores were consistent with histological observations: the control group had the highest scores, followed by moderate declines in the HFD model, and sharp decreases in both GTW and HFD + GTW models, reflecting profound impairment of spermatogenesis (Figure 2F).

### 3.3 GTW, HFD, and HFD + GTW Exposures Induced Gut Microbiota Dysbiosis With Distinct Patterns Linked to Reproductive Outcomes

**FIGURE 3:**
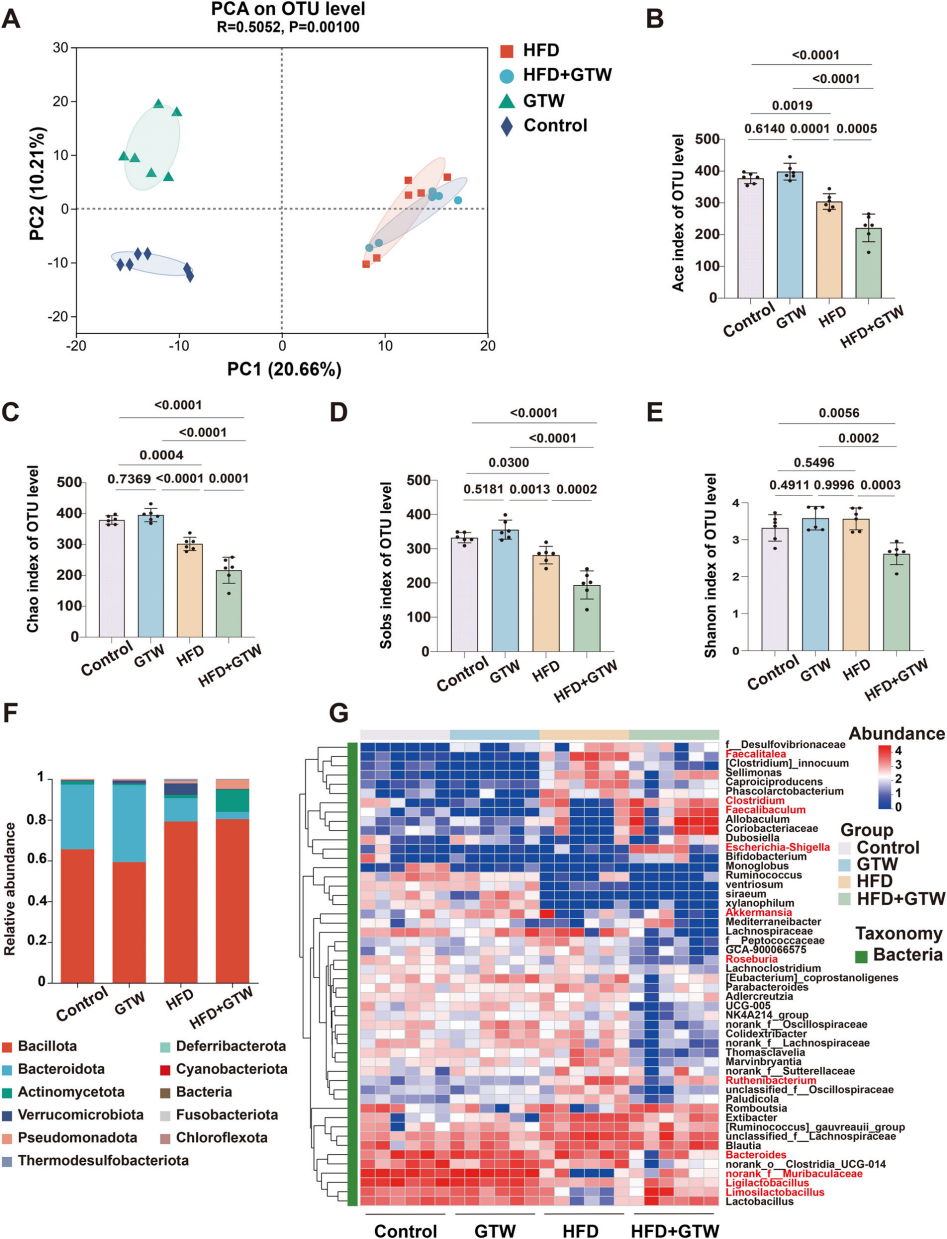
Gut microbial diversity and compositional shifts induced by HFD and GTW exposure. (A) Principal component analysis (PCA) revealed a distinct separation between the groups based on the gut microbial OTU profiles. (B–E) Alpha diversity indices (i.e., Ace, Chao, Shannon, and Sobs) were significantly decreased in the HFD and HFD + GTW models, indicating reduced microbial richness and evenness. (F) Relative abundance at the phylum level showing decreased *Bacteroidota* and increased Bacillota in the HFD and HFD + GTW models. (G) Heatmap of the top 50 most abundant genera demonstrates depletion of probiotics (e.g., *Limosilactobacillus*, *Roseburia*) and enrichment of potentially harmful genera (e.g., *Escherichia*–*Shigella*, *Faecalitalea*) in the obese and combined models. Data are presented as the mean ± SEM. Statistical significance was determined using one‐way anova with a post hoc test.

Hierarchical clustering analysis (Figure S2) and PCA demonstrated a clear separation between the fecal microbiota of HFD rats and those fed a standard diet, indicating that HFD significantly altered gut microbiota composition (Figure 3A). Alpha diversity analysis revealed that the richness indices (Ace, Chao1, and Sobs) were significantly lower in both the HFD and HFD + GTW models than in the control group. The Shannon index was significantly lower in the HFD + GTW model than in the control group, indicating an overall decline in microbial diversity, including both species richness and evenness. This decline suggests a shift toward microbial community imbalance, with dominance by fewer microbial taxa, a pattern typically associated with dysbiosis and impaired host health. All three richness indices (Ace, Chao1, and Sobs) were significantly lower in the HFD and HFD + GTW models than in the GTW model, indicating reduced species counts in the obese microbiomes. Notably, the combined HFD + GTW model exhibited the most severe decline, with the Shannon index significantly lower than that of both GTW and HFD models, reflecting an additive detrimental effect of GTW exposure on top of obesity (Figure 3B–E). At the phylum level, relative abundance analysis showed that *Bacteroidota* was significantly decreased, whereas *Firmicutes* was significantly increased in the HFD and HFD + GTW models compared with the control group (*p* < 0.05) (Figure 3F). At the genus level, heatmap analysis of the top 50 taxa revealed distinct compositional differences among the models. The control group exhibited high relative abundances of beneficial genera such as *Limosilactobacillus*, *Ligilactobacillus*, *Akkermansia*, and *Roseburia*.

In contrast, these probiotics were significantly depleted in the HFD and HFD + GTW models, while potentially pathogenic or inflammation‐associated genera, including *Escherichia–Shigella*, *Faecalibaculum*, *Clostridium*, *Ruthenibacterium*, and *Faecalitalea*, were enriched. Interestingly, the GTW model displayed a unique microbial pattern characterized by increased *Muribaculaceae* and *Bacteroides*‐related genera, while the reduction in probiotics was less pronounced compared with the HFD‐based models (Figure 3G).

**FIGURE 4:**
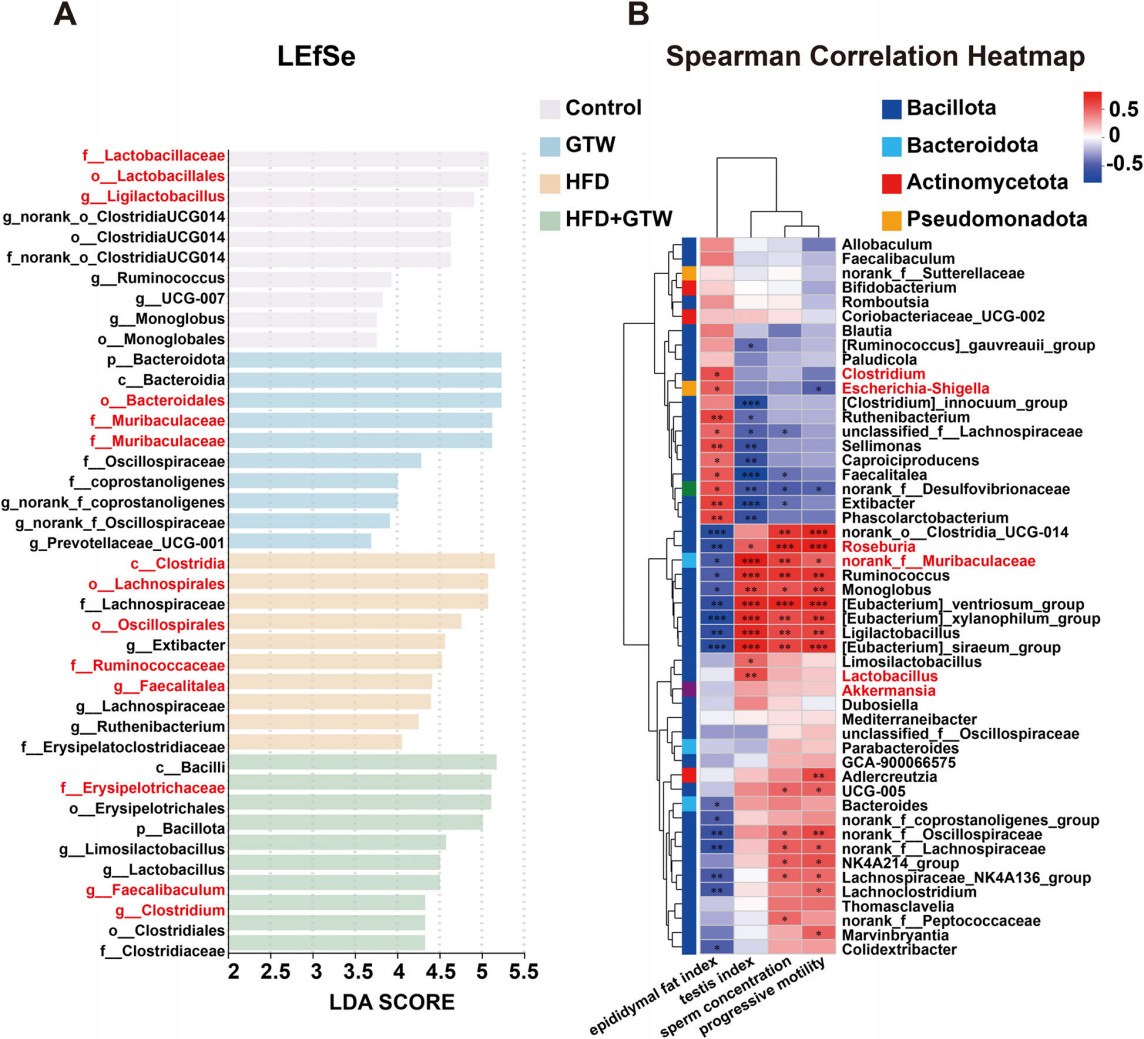
Differential microbial signatures and their associations with reproductive parameters. (A) LEfSe analysis (LDA score > 2.0, *p* < 0.05) identified the key discriminative taxa across groups. The control group rats were enriched in *Ligilactobacillus* and *Lactobacillaceae*, whereas the HFD + GTW model showed enrichment in *Faecalibaculum* and *Clostridium*. (B) Spearman correlation heatmap illustrated associations between specific genera and host parameters (i.e., epididymal fat index, testis index, sperm concentration, and progressive motility). Red and blue represent positive and negative correlations, respectively.

LEfSe analysis identified specific taxa with significant intergroup differences. The control group was enriched in *Lactobacillaceae*, *Lactobacillales*, and *Ligilactobacillus*, whereas the GTW model showed enrichment of *Muribaculaceae* and *Bacteroidales*. In contrast, the HFD model was dominated by *Clostridia*, *Lachnospiraceae*, *Oscillospirales*, *Ruminococcaceae*, and *Faecalitalea*, while the HFD + GTW model exhibited further enrichment of *Erysipelotrichaceae*, *Faecalibaculum*, and *Clostridium* (Figure 4A). Spearman correlation analysis between gut microbiota (at the genus level) and physiological parameters, including epididymal fat index, testis index, sperm concentration, and progressive sperm motility, revealed strong associations (Figure 4B). Color intensity signified the correlation coefficient (red: positive; blue: negative). As shown in Figure 4B, the epididymal fat index was positively correlated with *Clostridium*, *Escherichia–Shigella*, *Ruthenibacterium*, *Lachnospiraceae*, *Sellimonas*, *Caproiciproducens*, *Faecalitalea*, *Desulfovibrionaceae*, *Extibacter*, and *Phascolarctobacterium*, but negatively correlated with *Clostridia*, *Roseburia*, *Muribaculaceae*, *Ruminococcus*, *Monoglobus*, *Eubacterium* groups, *Ligilactobacillus*, *Bacteroides*, and several *Lachnospiraceae*‐related genera. The testis index showed positive correlations with *Roseburia*, *Muribaculaceae*, *Ruminococcus*, *Monoglobus*, *Eubacterium* groups, *Ligilactobacillus*, *Limosilactobacillus*, and *Lactobacillus*, and negative correlations with *Ruthenibacterium* and certain *Clostridium* and *Lachnospiraceae* genera. The sperm concentration was positively associated with *Clostridia*, *Roseburia*, *Muribaculaceae*, *Ruminococcus*, *Monoglobus*, and *Eubacterium* groups, but negatively correlated with *Desulfovibrionaceae*, *Faecalitalea*, and *Extibacter*. Progressive sperm motility was positively correlated with *Clostridia*, *Roseburia*, *Muribaculaceae*, *Ruminococcus*, *Monoglobus*, *Ligilactobacillus*, *[Eubacterium]_xylanophilum*, *[Eubacterium]_siraeum*, *Adlercreutzia*, *Oscillospiraceae*, *Lachnospiraceae*, *Lachnoclostridium*, and *Marvinbryantia*, whereas it was significantly negatively correlated with *Escherichia–Shigella* and *Desulfovibrionaceae*.

### 3.4 GTW, HFD, and HFD + GTW Exposures Resulted in Distinct Testicular Gene Expression Profiles

**FIGURE 5:**
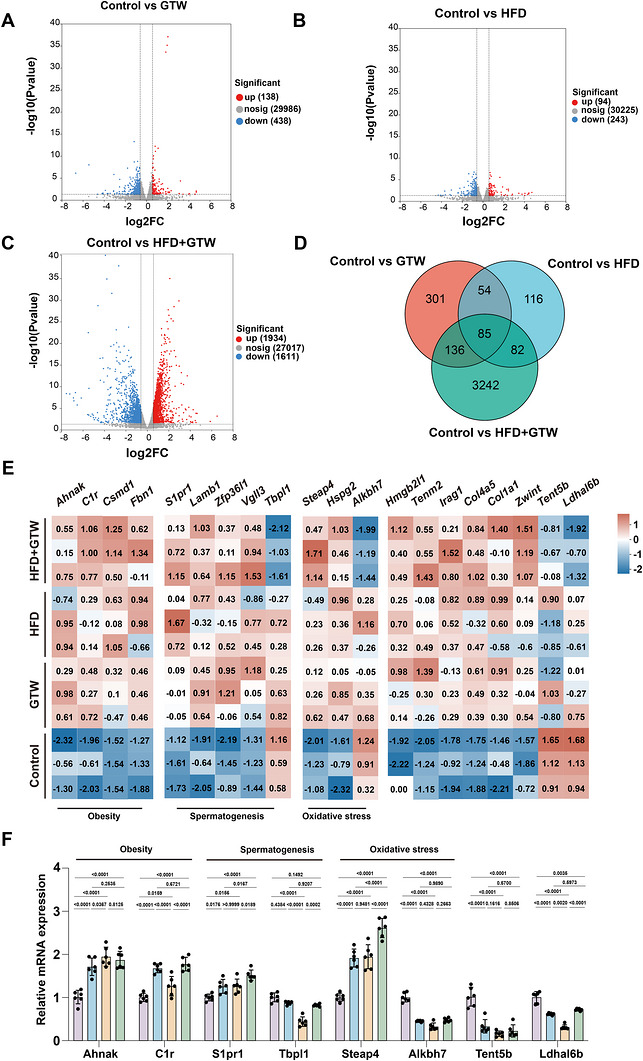
Transcriptomic alterations in testicular tissues following HFD and GTW exposure. (A–C) Volcano plots displaying DEGs between the control group and each model, exhibiting relatively more extensive changes in the HFD + GTW model. (D) Heatmap of top 20 shared DEGs across all model groups, based on the fold changes and adjusted *p* value. (E) Hierarchical clustering heatmap displaying representative DEGs grouped into four functional categories: obesity‐related (Ahnak, C1r, Csmd1, Fbn1), spermatogenesis‐related (S1pr1, Lamb1, Zfp36l1, Vgll3, Tbpl1), oxidative stress‐related (Steap4, Hspg2, Alkbh7), and genes without prior characterization in the reproductive or metabolic pathways (e.g., Hmgb2l1, Tenm2). Color intensity indicates the relative expressions (red: upregulation; blue: downregulation). The HFD + GTW group exhibited the most pronounced transcriptomic remodeling when compared with other models. (F) Relative mRNA expressions of eight representative genes from four functional categories: obesity‐related (*Ahnak*, *C1r*), spermatogenesis‐related (*S1pr1*, *Tbpl1*), oxidative stress‐related (*Steap4*, *Alkbh7*), and genes without prior characterization in reproductive or metabolic pathways (*Tent5b*, *Ldhal6b*), were assessed by qRT‐PCR across experimental groups. The results confirmed the transcriptomic trends, with *Ahnak*, *C1r*, *S1pr1*, and *Steap4* significantly upregulated in the model groups, particularly in the HFD + GTW rats, whereas *Alkbh7*, *Tbpl1*, *Tent5b*, and *Ldhal6b* were significantly downregulated. Data are presented as the mean ± SEM (*n* = 6/group). Statistical significance was determined by one‐way anova, followed by post hoc tests.

To elucidate the molecular mechanisms underlying GTW‐, HFD‐, and HFD + GTW‐induced oligoasthenozoospermia, RNA sequencing (RNA‐seq) analysis was performed on testicular tissues. DEGs were identified using a threshold of fold change of > 1.5 and *p* value of < 0.05. Distinct DEG patterns emerged across the experimental models. Compared with the control group, the GTW model exhibited 576 DEGs (438 upregulated and 138 downregulated; Figure 5A), while the HFD model showed 337 DEGs (243 upregulated, 94 downregulated; Figure 5B). In contrast, the HFD + GTW model demonstrated the most extensive transcriptomic alterations, with 3545 DEGs (1611 upregulated, 1934 downregulated; Figure 5C). Venn diagram analysis revealed 85 overlapping DEGs shared among all three models, representing a core set of genes consistently dysregulated under diverse pathological conditions. Notably, the HFD + GTW model had the largest number of unique DEGs (3242), far exceeding the GTW (301) and HFD (116) models, suggesting that combined exposure elicited a more comprehensive and complex transcriptional response (Figure 5D). A hierarchical clustering heatmap was created to visualize representative DEGs across all samples (Figure 5E). In this heatmap, each row represented a sample and each column represented a gene, with color intensity indicating the relative expression (red for upregulation and blue for downregulation). These genes were grouped into four functional categories: (1) obesity‐related (*Ahnak*, *C1r*, *Csmd1*, and *Fbn1*), (2) spermatogenesis‐related (*S1pr1*, *Lamb1*, *Zfp36l1*, *Vgll3*, and *Tbpl1*), (3) oxidative stress‐related (*Steap4*, *Hspg2*, and *Alkbh7*), and (4) uncharacterized or regulatory genes (*Tent5b* and *Zwint*). In the heatmap, the control group exhibited low expression of stress‐ and obesity‐associated genes, while GTW and HFD models showed moderate upregulation. The HFD + GTW model displayed extensive transcriptional remodeling, with strong upregulation of *Ahnak*, *C1r*, *S1pr1*, and *Steap4*, and downregulation of *Alkbh7*, *Tbpl1*, and *Ldhal6b*. Several spermatogenesis‐associated genes (*Zfp36l1* and *Vgll3*) were also elevated, whereas *Tent5b* and *Zwint* exhibited marked dysregulation. These changes suggest a synergistic impact of combined metabolic and toxicant stress on gene networks related to metabolism, germ cell function, and oxidative damage. qRT‐PCR analysis confirmed the RNA‐seq results, showing consistent upregulation of *Ahnak*, *C1r*, *S1pr1*, and *Steap4* and downregulation of *Alkbh7*, *Tent5b*, *Ldhal6b*, and *Tbpl1* across all models, with the most pronounced changes in the HFD + GTW model (Figure 5F). The high concordance between RNA‐seq and qRT‐PCR results confirms the reliability of the transcriptomic findings and underscores the functional importance of these genes in testicular stress responses.

**FIGURE 6:**
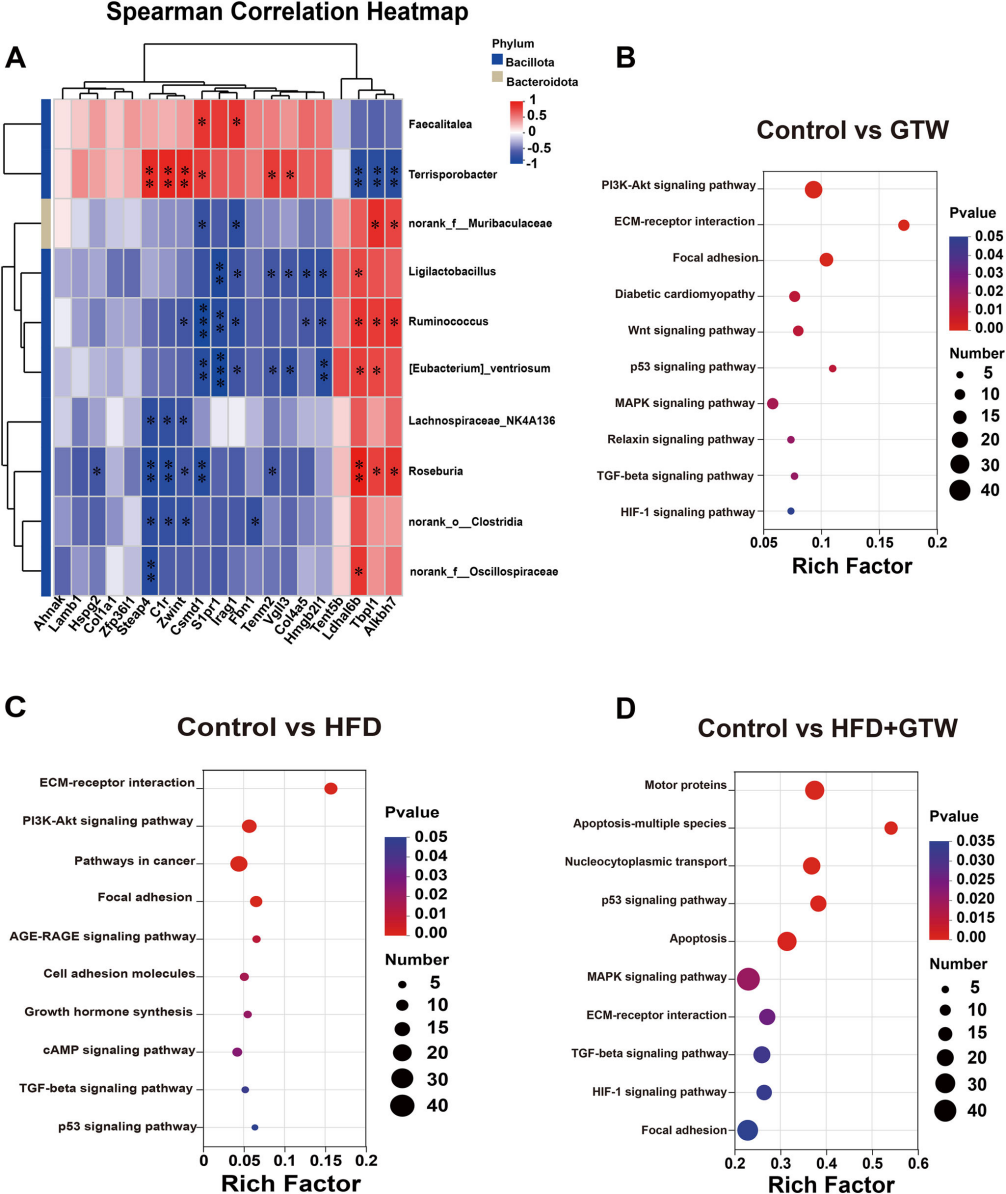
Associations between gut microbiota and testicular gene expressions and the enrichment of transcriptomic‐signaling pathways. (A) Spearman correlation heatmap showing associations between representative gut microbial genera (*Y*‐axis) and differentially expressed testicular genes (*X*‐axis). Red and blue indicate positive and negative correlations, respectively; asterisks denote statistical significance (*p < 0.05). Notably, *Faecalitalea* and *Terrisporobacter* were positively correlated with stress‐ and inflammation‐related genes (e.g., *Steap4*, *C1r*, *S1pr1*), whereas *Muribaculaceae*, *Ligilactobacillus*, and *Ruminococcus* were positively associated with spermatogenesis‐related genes (e.g., *Alkbh7*, *Tbpl1*). (B–D) KEGG pathway enrichment analysis of differentially expressed genes in the GTW, HFD, and HFD + GTW model groups compared with the control. Enriched pathways included PI3K‐Akt, ECM–receptor interaction, MAPK, apoptosis, and TGF‐beta signaling, with the HFD + GTW group exhibiting the broadest and most significant enrichment of stress‐related and apoptotic pathways. Dot size represents gene number; color indicates *p* value.

Furthermore, Spearman correlation heatmaps were created to explore the associations between gut microbiota (at the genus level) and the expressions of testis‐related genes (Figure 6A). The color intensity in the heatmap signifies the strength of the correlation coefficient (red and blue indicating positive and negative correlations, respectively). Many bacterial genera showed strong correlations with key genes involved in testicular function and spermatogenesis (Figure 6A). *Faecalitalea* and *Terrisporobacter* showed broad positive correlations with *Steap4*, *C1r*, *Zwint*, *Csmd1*, *S1pr1*, *Irag1*, *Tenm2*, and *Vgll3*. In contrast, beneficial genera such as *Muribaculaceae*, *Ligilactobacillus*, *Ruminococcus*, and *Ventriosum* exhibited significant negative correlations with *Csmd1*, *S1pr1*, *Irag1*, *Tenm2*, *Vgll3*, *Col4a5*, and *Hmgb2l1*. *Lachnospiraceae*, *Roseburia*, *Clostridia*, and *Oscillospiraceae* were negatively correlated with *Steap4*, *C1r*, and *Zwint*. Notably, *Roseburia*, *Ventriosum*, *Ruminococcus*, *Ligilactobacillus*, and *Muribaculaceae* were positively correlated with *Ldhal6b*, *Tbpl1*, and *Alkbh7*, whereas *Terrisporobacter* was negatively correlated with these genes. The functions of the DEGs were classified according to the GO analysis. The genes were enriched in the metabolic process category (Figure S3).

In the GTW model, DEGs were enriched in pathways related to cell signaling, extracellular matrix (ECM) remodeling, and stress responses (e.g., PI3K‐Akt, Wnt, MAPK, p53, and HIF‐1 signaling; Figure 6B). HFD‐induced DEGs were associated with immune regulation and adhesion pathways (e.g., ECM–receptor interaction, TGF‐β, AGE‐RAGE, and cAMP signaling; Figure 6C). In contrast, the HFD + GTW model displayed broader dysregulation encompassing apoptosis, stress responses, and ECM remodeling, with enrichment in MAPK, p53, HIF‐1, TGF‐β, and focal adhesion pathways. Overall, the combined HFD + GTW exposure induced the most severe and complex alterations, linking apoptosis, stress, and ECM disruptions beyond single exposures (Figure 6D).

## 4 Discussion

Globally, male fertility is in decline, with oligoasthenozoospermia identified as one of the principal contributing factors [20]. Understanding its pathological mechanisms and exploring targeted treatment strategies are therefore of considerable importance. In this study, we established three rat models of oligoasthenozoospermia: GTW, HFD, and HFD + GTW models. Our results demonstrated that all three models exhibited marked reductions in sperm concentration and motility, accompanied by evident testicular and epididymal histopathological damage. Among them, the HFD + GTW model showed the most pronounced impairments in sperm concentration, motility, and testicular architecture. The 16S rRNA‐seq data further confirmed that the rats in the GTW model exhibited a different microbial profile, characterized by elevated *Muribaculaceae* and *Bacteroides*‐related genera, whereas the HFD and HFD + GTW models exhibited depletion of beneficial genera and enrichment of potentially pathogenic taxa such as *Escherichia–Shigella* and *Faecalibaculum*. Transcriptomic profiling corroborated these findings, showing that GTW and HFD individually disrupted pathways associated with stress responses and tissue remodeling, while their combined exposure amplified apoptosis‐ and stress‐related signaling.

Both obesity and reproductive toxicants are recognized contributors to male infertility [21, 22], yet their pathogenic mechanisms remain incompletely understood, and current therapies are largely empirical. Hence, developing representative animal models is critical for clarifying disease mechanisms and identifying therapeutic targets. GTW is widely used in the clinical treatment of autoimmune and inflammatory disorders and can cause reproductive toxicity in men, leading to reduced sperm quality and, in severe cases, infertility if used for a long time [23, 24, 25, 26]. Thus, GTW‐induced oligoasthenozoospermia provides a relevant model for studying drug‐related reproductive toxicity and its implications for safe clinical use. In our models, GTW exposure did not significantly alter body weight, BMI, obesity index, or lipid parameters, whereas both the HFD and HFD + GTW models developed significant metabolic abnormalities, including dyslipidemia characterized by elevated TC, TG, and LDL‐C and decreased HDL‐C levels. These results confirm that the metabolic disturbances observed in the combined model were primarily attributable to HFD rather than GTW exposure.

Serum biochemical parameters indicated that hepatic transaminases (AST and ALT) remained within the normal range in all models, while serum Cr levels in the HFD + GTW model were slightly elevated compared with the control group but still within physiological limits [27], suggesting that neither GTW nor HFD exposure caused overt hepatic or renal dysfunction. Despite this, the testicular tissue showed significant spermatogenic impairment, manifested as reduced sperm density (< 15 × 10⁶/mL), decreased progressive motility (< 32%), and marked declines in total sperm count and motility. These functional deficits coincided with endocrine dysregulation, including reduced T and LH and elevated FSH levels. Although all three models replicated key features of oligoasthenozoospermia, the extent of impairment severity was inconsistent, with the HFD + GTW model exhibiting the greatest testicular damage, lowest sperm quality indices, and most disorganized testicular architecture. Importantly, the absence of systemic hepatic or renal toxicity indicates that these reproductive impairments were not secondary to generalized organ damage but were likely the result of direct testicular effects of metabolic and toxic stress. GTW toxicity can affect both hepatic and reproductive systems, but its severity depends strongly on dose, exposure duration, and experimental conditions [28]. While high doses (e.g., 270 mg/kg) induce liver enzyme elevation [29], the moderate dose used in this study (60 mg/kg) primarily targeted the reproductive system without producing overt hepatic injury.

To systematically elucidate the complex pathological mechanisms underlying oligoasthenozoospermia, we conducted a comprehensive multiomics analysis integrating transcriptomic profiling and 16S rRNA‐seq. The gut microbiota of rats exposed to different oligoasthenozoospermia‐inducing factors exhibited distinct compositional alterations at both the phylum and genus levels. Notably, rats in the HFD and HFD + GTW models showed an increased relative abundance of *Firmicutes* and a decreased abundance of *Bacteroidota* compared with the control group, resulting in an elevated *Firmicutes/Bacteroidetes* (F/B) ratio. The F/B ratio is widely recognized as an indicator of gut microbial imbalance [30], and shifts in this ratio have been linked to metabolic dysfunction and reproductive disorders [31]. In this study, alterations in *Firmicutes* and *Bacteroidota* abundance, along with the increased F/B ratio in the HFD and HFD + GTW models, suggest that obesity‐ and toxicant‐induced oligoasthenozoospermia are associated with gut microbiota dysbiosis, which may contribute to reductions in sperm concentration and motility. At the genus level, both heatmap and LEfSe analyses consistently demonstrated that HFD and HFD + GTW exposure significantly disrupted gut microbial homeostasis. These changes were characterized by a decline in beneficial genera such as *Lactobacillus*, *Akkermansia*, and *Roseburia*, alongside an enrichment of potentially pathogenic taxa, including *Escherichia–Shigella* and *Clostridium*. The alterations were most pronounced in the HFD + GTW model, indicating a synergistic exacerbation of gut microbiota dysbiosis under combined metabolic and toxicant stress [32, 33, 34]. Correlation analyses further revealed that *Lactobacillus*, *Akkermansia*, and *Roseburia* were positively correlated with testis index and sperm quality, whereas *Escherichia–Shigella* and *Clostridium* were negatively correlated with these parameters, implying a close link between gut microbiota dysbiosis and impaired spermatogenesis [35]. Interestingly, the GTW model presented a distinct microbial signature dominated by *Bacteroidota*, particularly *Muribaculaceae* and related taxa. Correlation analysis demonstrated that *Muribaculaceae* abundance was positively associated with testis index and sperm quality, consistent with previous findings [36], suggesting a potential compensatory or protective microbial adaptation in response to toxicant‐induced stress. Although the overall microbial diversity in the GTW model remained relatively stable compared with the control group, shifts in dominant bacterial taxa indicate that reproductive toxicants can selectively alter gut microbial composition. Collectively, these findings support the concept that gut microbiota dysbiosis contributes to the pathogenesis of oligoasthenozoospermia through the “gut–testis axis,” providing a mechanistic basis for developing microbiota‐targeted therapeutic interventions.

Subsequent RNA‐seq analysis revealed a distinct transcriptional separation between the control and all three models, as shown by hierarchical clustering of DEGs. Venn diagram analysis further clarified the shared and unique transcriptional responses among the models. A total of 85 DEGs were consistently altered across all models, suggesting a core gene set that may mediate the common pathological responses to reproductive insults. To better understand these mechanisms, the DEGs were categorized into four functional groups: obesity‐related, spermatogenesis‐related, oxidative stress‐related, and uncharacterized genes, and their potential roles in testicular dysfunction were examined. These findings were subsequently validated by RT‐qPCR. Obesity‐related genes such as *Ahnak*, *C1r*, and *Csmd1* were consistently upregulated across all models, with the most pronounced changes in the HFD + GTW model. *Ahnak* has been associated with obesity‐induced inflammation and metabolic dysregulation by promoting adipocyte hypertrophy and insulin resistance [37]. *C1r*, a component of the classical complement pathway, may exacerbate adipose inflammation and endocrine imbalance, indirectly impairing spermatogenesis [38]. *Csmd1* has been linked to BMI and lipid traits in genome‐wide association studies, implicating its role in lipid metabolism and systemic inflammation [39]. Together, these findings suggest that adipose‐derived inflammatory mediators contribute to testicular dysfunction in combined obesity–toxicant conditions. Spermatogenesis‐related genes, including S1pr1, Zfp36l1, and Vgll3, were markedly upregulated. *S1pr1*, a receptor involved in lipid signaling and immune modulation, increases oxidative stress and compromises blood–testis barrier integrity under stress conditions [40, 41]. *Zfp36l1*, which regulates mRNA stability and cell cycle progression, may inhibit spermatogonial proliferation when aberrantly expressed [42]. *Vgll3* acts as a negative regulator of Sertoli cell proliferation and testis development; its upregulation reflects impaired Sertoli–germ cell support, ultimately hindering spermatogenesis [43]. These transcriptional changes point toward suppressed germ cell development rather than compensatory regeneration. Within the oxidative stress‐related gene group, Steap4 and Hspg2 were significantly upregulated, while Alkbh7 was downregulated. *Steap4* promotes iron reduction and reactive oxygen species formation, contributing to oxidative damage in metabolic tissues [44]. *Hspg2* (perlecan), typically involved in maintaining ECM integrity, can aggravate endoplasmic reticulum stress and endothelial dysfunction when overexpressed under oxidative conditions [45]. Conversely, *Alkbh7*, a mitochondrial demethylase that supports oxidative metabolism, was downregulated, indicating impaired mitochondrial function and compromised redox balance [46]. Interestingly, genes with limited prior association to reproduction, such as *Zwint*, *Tent5b*, and *Tbpl1*, were consistently dysregulated in all models. Their expression correlated with both sperm quality parameters and shifts in gut microbial taxa, highlighting their potential roles as previously unrecognized regulators within the gut–testis axis. Further studies are warranted to define their specific contributions to testicular homeostasis and host–microbiota interactions. In summary, dysregulation across these four functional gene groups underscores a multifaceted pathogenic process in which metabolic stress, inflammation, oxidative injury, and gut microbiota imbalance converge to impair spermatogenesis in obesity‐ and toxicant‐induced oligoasthenozoospermia.

Interestingly, Spearman correlation analysis revealed strong interconnections among gut microbiota composition, testicular gene expression, and key reproductive parameters. Several microbial genera altered by HFD and HFD + GTW exposure were significantly associated with DEGs linked to oxidative stress, inflammation, and metabolic dysfunction. Specifically, *Faecalitalea* and *Terrisporobacter*, which were enriched in the HFD and HFD + GTW models, were positively correlated with upregulated stress‐related genes such as *Steap4*, *C1r*, *S1pr1*, and *Csmd1*. These genera were simultaneously negatively correlated with sperm concentration, progressive motility, and testis index, indicating that their overgrowth may promote testicular injury by driving proinflammatory and stress‐associated transcriptional programs. Conversely, beneficial taxa such as *Muribaculaceae*, *Ligilactobacillus*, *Ruminococcus*, and *Roseburia* were positively correlated with genes involved in spermatogenic maintenance and metabolic balance, including *Alkbh7*, *Ldhal6b*, and *Tbpl1*, and negatively correlated with inflammatory and stress‐related genes. These taxa were also positively correlated with higher sperm quality indices and testis weight, suggesting protective roles in preserving testicular homeostasis. Collectively, these findings reveal a potential mechanistic cascade in which gut microbiota dysbiosis, characterized by the depletion of protective bacteria and expansion of proinflammatory genera, modulates testicular gene expression and contributes to functional decline of the reproductive axis. This integrative evidence supports the hypothesis that microbiota disturbances promote oligoasthenozoospermia via the gut–testis axis and underscores the strength of our multiomics approach in uncovering this interplay. While single‐factor models (GTW and HFD) triggered moderate activation of oxidative stress and metabolic dysfunction pathways, these effects were markedly amplified in the combined HFD + GTW model, as confirmed by KEGG pathway enrichment analysis. In the control versus GTW comparison, DEGs were enriched in canonical stress and structural regulation pathways, including PI3K‐Akt, ECM–receptor interaction, MAPK, HIF‐1, and p53 signaling, reflecting the cellular response to reproductive toxicants. Similarly, control group versus HFD model DEGs were enriched in overlapping pathways, along with AGE‐RAGE and cAMP signaling, implicating obesity‐induced metabolic and inflammatory stress. Notably, the control group versus HFD + GTW model comparison exhibited the broadest and most profound dysregulation, with enrichment of apoptosis, nucleocytoplasmic transport, motor protein regulation, and elevated activation scores in MAPK, HIF‐1, TGF‐β, and p53 pathways. These findings point to synergistic cellular damage involving apoptosis, oxidative stress, and cytoskeletal disruption. Together, these results indicate that while reproductive toxicity and obesity each perturb stress‐response and structural integrity pathways individually, their coexistence intensifies both the extent and complexity of molecular dysregulation. The pronounced activation of apoptosis‐ and stress‐related signaling in the HFD + GTW model explains the more severe spermatogenic damage observed histologically and functionally. Overall, these transcriptomic and pathway‐level alterations highlight oxidative stress, inflammation, and ECM remodeling as central mechanisms driving oligoasthenozoospermia, particularly under the combined influence of metabolic and toxicant stress.

In summary, this study established a series of preclinical rat models that reproduce distinct etiological pathways underlying oligoasthenozoospermia, namely, reproductive toxicity, obesity, and their combined effects. Among these, the HFD + GTW model demonstrated a uniquely severe and multifactorial phenotype that clearly distinguished it from either single‐exposure model. Reproductively, this combined model exhibited the most pronounced impairments in sperm quality, testicular architecture, and hormonal regulation. At the microbiota level, it showed the greatest loss of alpha diversity, a marked depletion of beneficial bacterial taxa, and a notable enrichment of proinflammatory genera, indicating a synergistic disruption of gut microbial homeostasis. Transcriptomically, the HFD + GTW model displayed the broadest molecular perturbations, with the highest number of DEGs and strong activation of stress‐related pathways, including apoptosis, oxidative stress, and ECM remodeling. Importantly, several microbial shifts in the HFD + GTW model were strongly correlated with altered expression of key testicular genes, suggesting a microbiota‐mediated contribution to reproductive dysfunction. Collectively, these integrated findings confirm the HFD + GTW model as a representative and clinically relevant platform for studying reproductive impairment under combined metabolic and toxicant stress.

These results also emphasize the need for clinical caution when administering GTW to overweight men or male patients with obesity, given the potential for compounded reproductive risks. By reproducing major pathological features of human male infertility, including testicular damage, hormonal disruption, and transcriptomic alterations, these models provide a robust and versatile framework for investigating the mechanisms of oligoasthenozoospermia and for developing targeted therapeutic interventions across different pathogenic contexts.

## Author Contributions

**Yunlong Yao**: Writing – original draft. **Xi Zhang**: Writing – original draft, Data curation. **Shuo Huang**: Writing – original draft, Conceptualization. **Yulin Zong**: Writing – original draft, Methodology. **Yunzhi Zhang**: Methodology, Data curation. **Mingrui Li**: Methodology, Validation. **Fufangyu Zhao**: Data curation, Validation. **Linghui Zhu**: Formal analysis. **Tianxing Li**: Data curation. **Lingru Li**: Writing – review and editing, Supervision. **Wenlong Sun**: Writing – review and editing, Supervision. **Mingyan Shao**: Writing – review and editing, Conceptualization, Project administration. **Yanfei Zheng**: Writing – review and editing, Supervision, Funding acquisition.

## Funding

This work was supported by the National Natural Science Foundation of China (Nos. 82174389 and 82474330, China), the High‐level Key Discipline of National Administration of Traditional Chinese Medicine‐Traditional Chinese Constitutional Medicine (No. zyyzdxk‐2023251).The Horizontal Project of Zhongshan Hospital of Traditional Chinese Medicine: Experimental Study on Regulating Phlegm‐Dampness Constitution for the Prevention and Treatment of Obesity with Oligoasthenospermia, BUCM‐2024‐JS‐FW‐059.

## Ethics Statement

SD rats' experiments were approved by the Ethics Committee for Laboratory Animals at Beijing University of Chinese Medicine (BUCM‐2024080504‐3077).

## Conflicts of Interest

The authors declare no conflicts of interest.

## Supporting information




**Figure S1**: Effects of GTW, HFD, and the combined exposure on testicular morphology, organ indices, hormone levels, and sperm motility. (A) Representative gross images of the testis and epididymis from each study group. (B, C) Testis organ index and epididymal fat index were significantly reduced in the HFD and HFD + GTW groups relative to those in the control group. (D–F) Evaluation of the serum levels of LH, estradiol (E2), and prolactin (PRL) showed: LH was significantly decreased in the HFD and HFD + GTW groups, whereas E2 and PRL showed no significant changes. (G) Total sperm motility was markedly reduced in all model groups, especially in the HFD + GTW group. Data are presented as the mean ± SEM (*n* = 8/group). One‐way anova with post hoc test was performed to assess the statistical significance of the data.
**Figure S2**: Hierarchical clustering of the gut microbiota composition across experimental groups. Hierarchical clustering tree based on operational taxonomic unit (OTU) level profiles of fecal microbiota in the rats from the control, GTW, HFD, and HFD + GTW groups. The clustering patterns display distinct microbial community structures among the groups, with the HFD and HFD + GTW samples forming separate branches from the control and GTW groups.
**Figure S3**: GO enrichment analysis of differentially expressed genes (DEGs) in testicular tissues. (A–C) Gene Ontology (GO) enrichment analysis (level 4) of DEGs between the control and GTW (A), HFD (B), or HFD + GTW (C) model groups. GO terms were classified into three categories: biological process (green), cellular component (red), and molecular function (blue). The bar length represents the number of DEGs associated with each term. The enriched terms included those involved in metabolic regulation, intracellular component organization, and stress responses, particularly under HFD + GTW exposure.
**Table S1**: Criteria employed for Johnsen scoring of seminiferous tubule cross‐sections.
**Table S2**: Characteristics of the primers designed for the target genes in real‐time PCR.

## Data Availability

Data will be made available upon request.
