# Supplementary material for: Comprehensive Quantification of Oligoasthenozoospermia Induced by Obesity, Reproductive Toxicants, and Their Combination in Rat Models
Source: Andrology. 2026 Jan 23;14(6):1535–51. doi: 10.1111/andr.70171 (PMC13432579; doi:10.1111/andr.70171)
Supplement: Supplementary file 1 — Figure S1: Effects of GTW, HFD, and the combined exposure on testicular morphology, organ indices, hormone levels, and sperm motility. (A) Representative gross images of the testis and epididymis from each study group. (B, C) Testis organ index and epididymal fat index were significantly reduced in the HFD and HFD + GTW groups relative to those in the control group. (D–F) Evaluation of the serum levels of LH, estradiol (E2), and prolactin (PRL) showed: LH was significantly decreased in the HFD and HFD + GTW groups, whereas E2 and PRL showed no significant changes. (G) Total sperm motility was markedly reduced in all model groups, especially in the HFD + GTW group. Data are presented as the mean ± SEM (n = 8/group). One‐way anova with post hoc test was performed to assess the statistical significance of the data. Figure S2: Hierarchical clustering of the gut microbiota composition across experimental groups. Hierarchical clustering tree based on operational taxonomic unit (OTU) level profiles of fecal microbiota in the rats from the control, GTW, HFD, and HFD + GTW groups. The clustering patterns display distinct microbial community structures among the groups, with the HFD and HFD + GTW samples forming separate branches from the control and GTW groups. Figure S3: GO enrichment analysis of differentially expressed genes (DEGs) in testicular tissues. (A–C) Gene Ontology (GO) enrichment analysis (level 4) of DEGs between the control and GTW (A), HFD (B), or HFD + GTW (C) model groups. GO terms were classified into three categories: biological process (green), cellular component (red), and molecular function (blue). The bar length represents the number of DEGs associated with each term. The enriched terms included those involved in metabolic regulation, intracellular component organization, and stress responses, particularly under HFD + GTW exposure. Table S1: Criteria employed for Johnsen scoring of seminiferous tubule cross‐sections. Table S2: Characteristics of the p [file ANDR-14-1535-s001.docx]

**Comprehensive Quantification of Oligoasthenozoospermia Induced by Obesity, Reproductive Toxicants, and their Combination in Rat Models**

**Supplementary Figures**


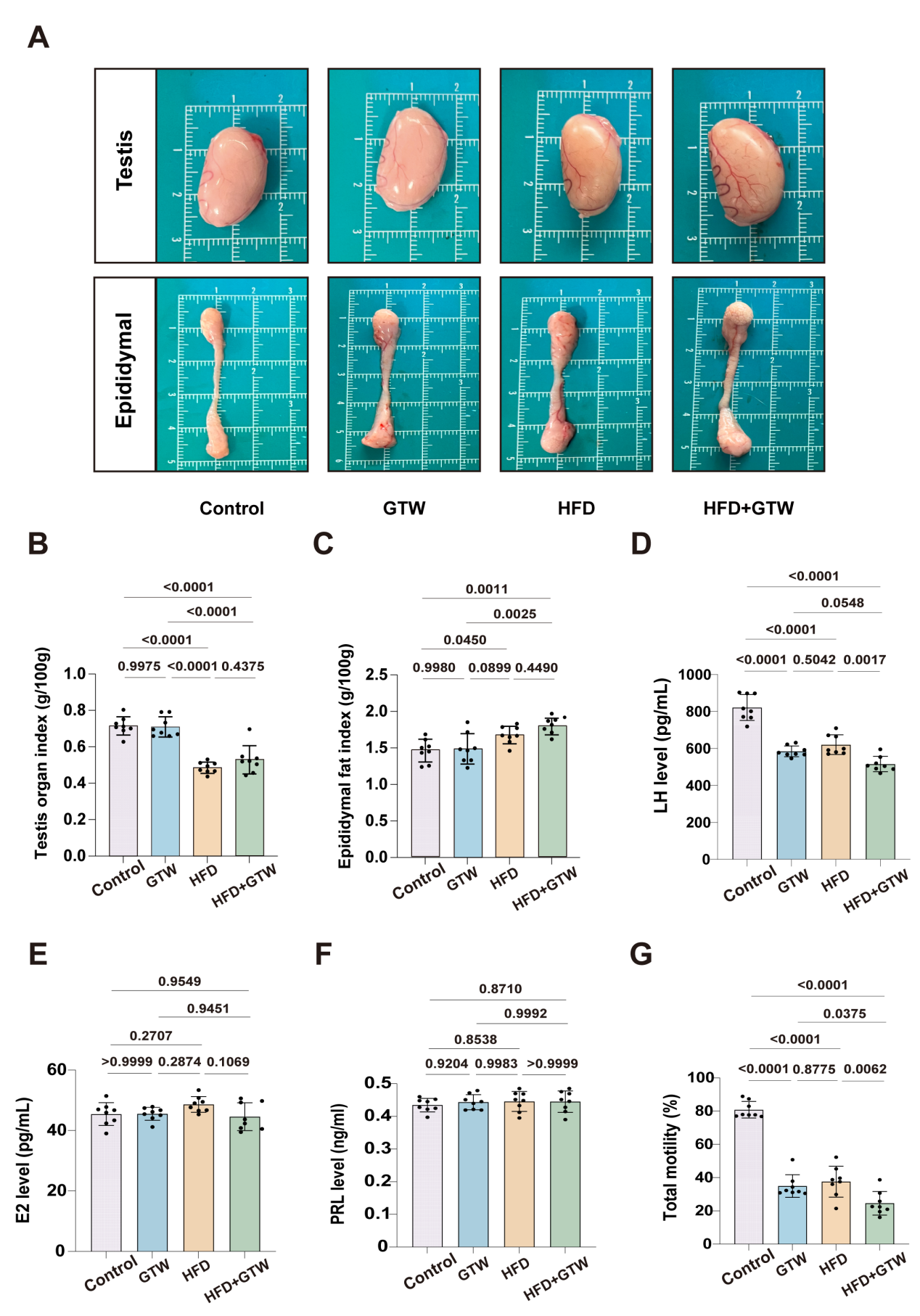


**Fig. S1. Effects of GTW, HFD, and the combined exposure on testicular morphology, organ indices, hormone levels, and sperm motility.**

1. Representative gross images of the testis and epididymis from each study group. (B–C) Testis organ index and epididymal fat index were significantly reduced in the HFD and HFD+GTW groups relative to those in the control group. (D–F) Evaluation of the serum levels of luteinizing hormone (LH), estradiol (E2), and prolactin (PRL) showed: LH was significantly decreased in the HFD and HFD+GTW groups, whereas E2 and PRL showed no significant changes. (G) Total sperm motility was markedly reduced in all model groups, especially in the HFD+GTW group. Data are presented as the mean ±SEM (n = 8/group). One-way ANOVA with post-hoc test was performed to assess the statistical significance of the data.


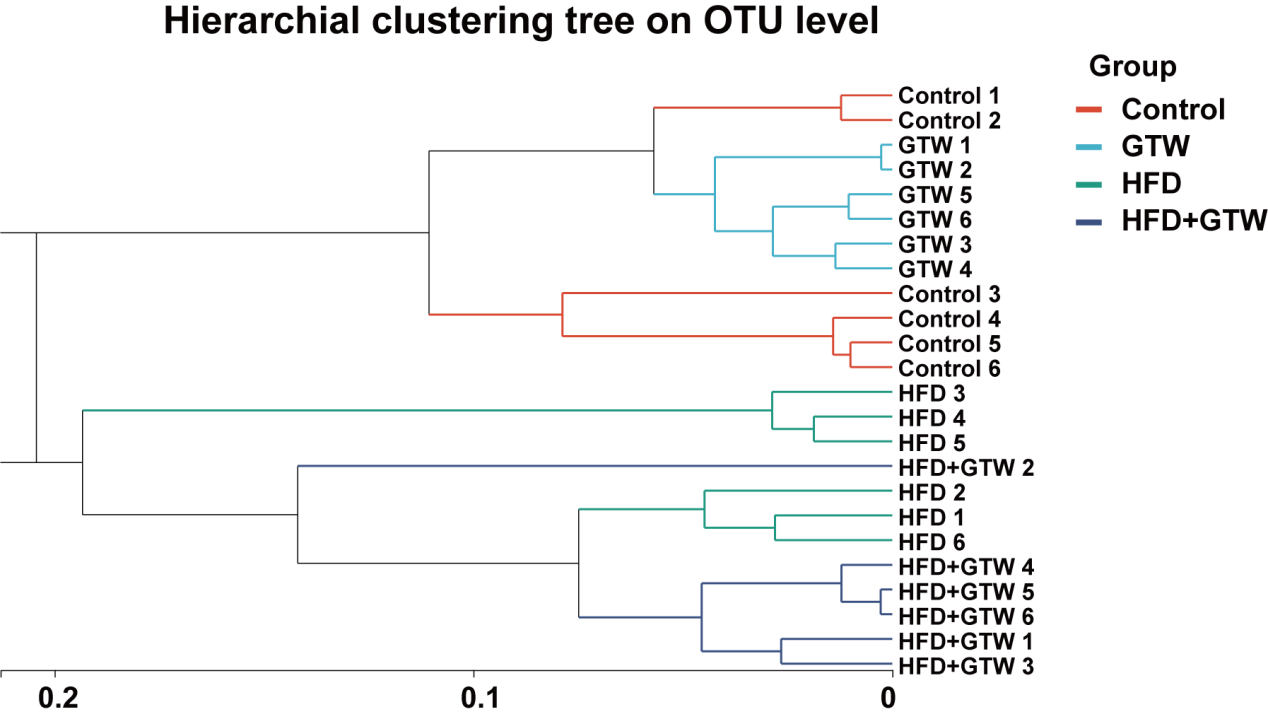


**Fig. S2. Hierarchical clustering of the gut microbiota composition across experimental groups.**

Hierarchical clustering tree based on operational taxonomic unit (OTU) level profiles of fecal microbiota in the rats from the control, GTW, HFD, and HFD+GTW groups. The clustering patterns display distinct microbial community structures among the groups, with the HFD and HFD+GTW samples forming separate branches from the control and GTW groups.


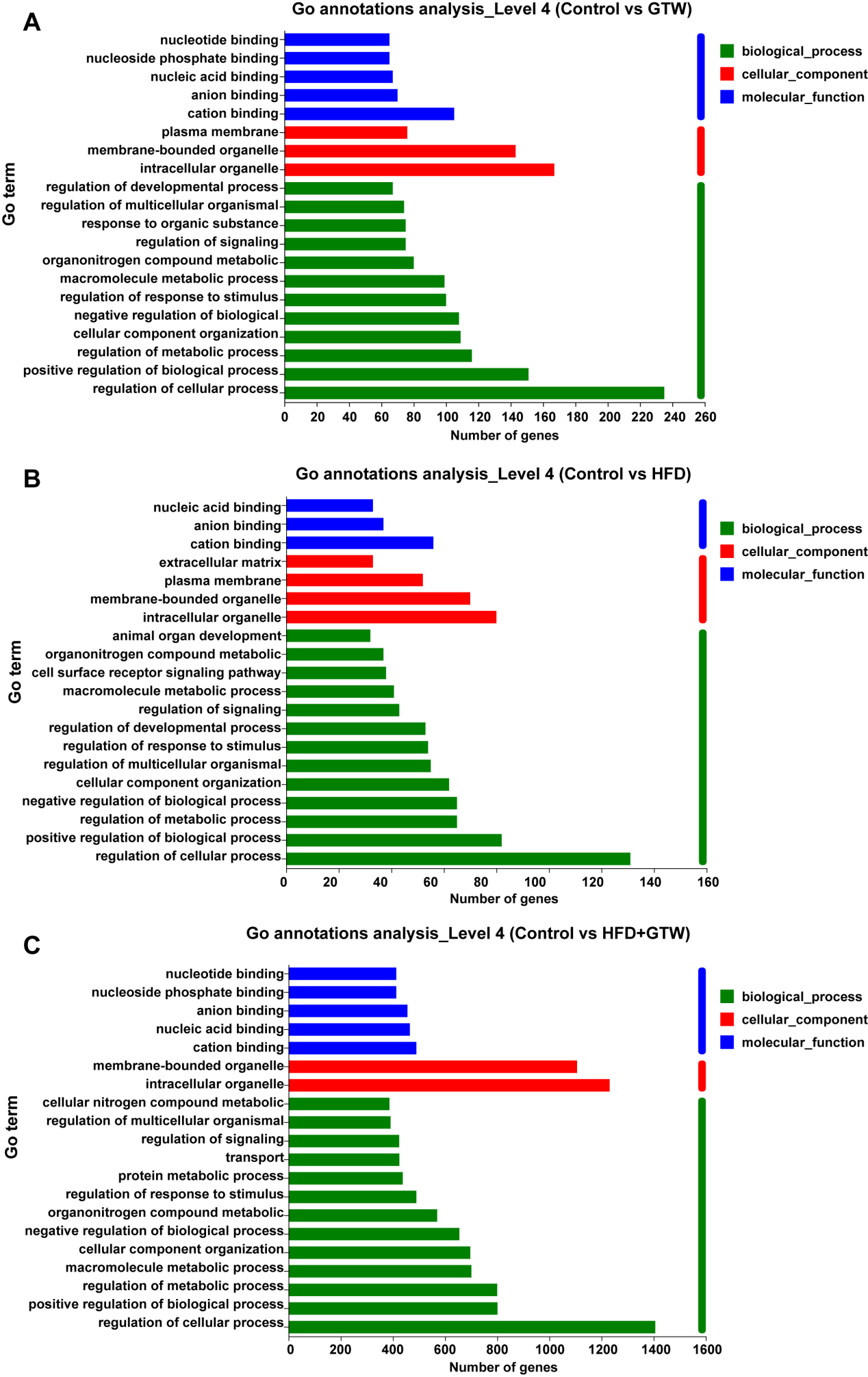


**Fig. S3. GO enrichment analysis of differentially expressed genes in testicular tissues.**

(A–C) Gene Ontology (GO) enrichment analysis (level 4) of differentially expressed genes (DEGs) between the control and GTW (A), HFD (B), or HFD+GTW (C) model groups. GO terms were classified into three categories: biological process (green), cellular component (red), and molecular function (blue). The bar length represents the number of DEGs associated with each term. The enriched terms included those involved in metabolic regulation, intracellular component organization, and stress responses, particularly under HFD+GTW exposure.

**Table S1 Criteria employed for Johnsen scoring of seminiferous tubule cross-sections.**

| Condition | Score |
| --- | --- |
| Absence of germ cells or Sertoli cells | 1 |
| Absence of germ cells | 2 |
| Presence of only spermatogonia | 3 |
| Presence of only a few spermatocytes | 4 |
| Absence of spermatozoa or spermatids, but presence of many spermatocytes | 5 |
| Presence of only a few spermatids | 6 |
| Absence of spermatozoa, but presence of many spermatids | 7 |
| Presence of only a few spermatozoa | 8 |
| Presence of many spermatozoa, but disorganized spermatogenesis | 9 |
| Complete spermatogenesis | 10 |

**Table S2 Characteristics of the primers designed for the target genes in real-time PCR**

| **Genes** | **Sequence (5′ – 3′)** |
| --- | --- |
| ***Ahnak*** | **TCAAGATACCGAGGCACGAAG**  **TGGTCCTGAGGCAGAAATGG** |
| ***C1r*** | **GAGTACCCGAAGCCCTATCCA**  **ATTCACCCAAGTTCTTCCCATTAG** |
| ***S1pr1*** | **CTTCATCAGGATCATATCTTGTTGC**  **GGTGGGAGGAGTTGTCTGATTT** |
| ***Tbpl1*** | **GGAAGATTGCTTTGGAGGGA**  **GCAAACGGATTTCAAAGGGC** |
| ***Steap4*** | **CTTCACGCCATCTACACCCTT**  **TGAGCCAGGCAGTAGAGGAAC** |
| ***Alkbh7*** | **GGCTTCAGGGAGACAGAGAAAT**  **ATAACACTTGGAGACAACAGGGAAA** |
| ***Tent5b*** | **CTGGACCTTATTACCATGCTAGCC**  **CATGGGGGTCACATAGTAATTAACG** |
| ***Ldhal6b*** | **CCATCAGCATCATAGCGAAAG**  **CCCGCTGTGATAACCACTACCT** |
